# Impact of Event Scale-6 (IES-6) for U.S. adults who experienced the COVID-19 pandemic

**DOI:** 10.1186/s12888-022-04136-2

**Published:** 2022-07-22

**Authors:** Jiin Jeong, Ah-Ram Kim, Claudia Hilton, Ickpyo Hong

**Affiliations:** 1 Department of Occupational Therapy, Graduate School, Yonsei University, 135 Backun Hall, 1 Yonseidae-gil, Wonju-si, Gangwon-do Republic of Korea; 2 Department of Occupational Therapy, University of Texas Medical Branch, Galveston, TX USA; 3 Department of Occupational Therapy, College of Software and Digital Healthcare Convergence, Yonsei University, 135 Backun Hall, 1 Yonseidae-gil, Wonju-si, Gangwon-do Republic of Korea

**Keywords:** COVID-19, Coronavirus, Impact of event scale-6, Post-traumatic stress disorder, Rasch analysis, Psychometrics

## Abstract

**Background:**

COVID-19 pandemic causes psychological problems such as stress. It is important to accurately identify the level of stress and establish effective intervention. The Impact of Event Scale-6 (IES-6) is widely used for post-traumatic stress disorder (PTSD) screening by measuring the level of subjective stress, but there has been no research on its psychometric properties with individuals who experienced the COVID-19 pandemic.

**Methods:**

A random sample of 600 participants were randomly selected from a COVID-19 survey database (*n* = 6391). Rasch analysis was conducted to examine item fit, rating scale structure, construct validity, differential item functioning (DIF), and precision of the IES-6.

**Results:**

The principal component analysis of Rasch residuals (54.1% of the raw variance explained) and the average of residual correlations (average *r* = .19) supported the unidimensionality structure in the IES-6. The rating scale was suitable, and the item difficulty hierarchy was logical. The item fit and the DIF contrast were acceptable, except for item 5. The IES-6’s person reliability was .76, which was also an acceptable level.

**Conclusions:**

This study showed that the IES-6 has acceptable item-level psychometrics for screening the stress level in adults in the United States for individuals who have experienced the COVID-19 pandemic. The findings suggested that the IES-6 would be useful for the rapid identification of the high-level stressand allow clinicians to quickly provide interventions for people with the COVID-19 related stress and their families.

**Supplementary Information:**

The online version contains supplementary material available at 10.1186/s12888-022-04136-2.

## Introduction

Coronavirus disease (COVID-19) was first identified in Wuhan, China, and the World Health Organization declared the epidemic on March 11, 2020 due to increased human-to-human transmission [1]. COVID-19 has a very high infection rate and a relatively high mortality rate (3.6 and 1.5% in and outside of China, respectively [2]). Because the disease affects people of all age groups, widespread infection prevention policies, such as “social distancing” and “shelter in place” have been adopted [3, 4].

Pandemic-related issues, such as social distancing and isolation, have amplified the fear of stigma in some cases [5]. Such isolation can have a detrimental effect on an individual’s physical and mental health [6]. It can also cause health problems other than COVID-19 infections, such as psychological pain and fear [7], and mental health problems (e.g., depression, stress, panic, distress, etc.) can lead to suicide accidents, suicide attempts, and actual suicide occurrences [8].

COVID-19 affects both physical and mental health [9]. Disasters such as the COVID-19 pandemic often lead to the diagnosis of mental disorders, such as post-traumatic stress disorder (PTSD), adjustment disorders, anxiety disorders, non-specific somatic symptoms, and substance abuse [10, 11]. In particular, PTSD negatively affects physical and mental health and increases demand for medical services [10].

A prior study showed that the difference between positive and negative emotions increased after the COVID-19 pandemic by the presence of anxiety, depression, and anger, while positive emotions and life satisfaction decreased [12]. Also, at the individual level, people are more likely to experience fear, helplessness, and stigma of getting sick or dying on their own [13]. PTSD symptoms are associated with long-lasting reduction in the ability to perform daily life activities [14] and reduction in quality of life [15], which are serious consequences for survivors and their families [16]. Therefore, clinical detection of PTSD symptoms is essential to be aware of potential consequences in well-being and quality of life.

Identifying the psychological problems that need to be addressed to help protect well-being and psychological health under COVID-19 is of paramount importance [17]. The accurate diagnosis of psychiatric syndromes associated with COVID-19 is an important first step toward best practice. It guides clinicians to choose the most appropriate and effective treatment and helps to be aware of potential prognoses [18]. A valid and reliable brief screening instrument for PTSD can be a valuable first stage in this process.

The Impact of Event Scale (IES)-6 consists of six items (total point range: 0–24) to quantify PTSD symptoms [19]. The IES-6 was developed based on the widely used Impact of Event Scale-Revised (IES-R). The validity and reliability of the IES-6 have been well evaluated [20]. However, information on the psychometric properties of the scale has yet been examined. Therefore, the objective of this study is to assess the internal consistency, criterion validity, and external construction validity of the IES-6 through Rasch analysis.

## Methods

### Participants

This study utilized the survey data from “Knowledge, attitudes, and practices related to COVID-19 in the U.S.,” registered in the Inter-university Consortium for Political and Social Research (ICPSR) [21]. This national survey sought to assess the state of COVID-19-related knowledge, beliefs, mental health, substance use changes, and behaviors among a sample of adults aged 18 years or older currently residing in the United States. The survey was administered online from March 20–30, 2020. Depression and anxiety were assessed using the Patient Health Questionnaire-4, stress was assessed using the Impact of Event Scale-6, and pessimism and changes in tobacco and alcohol use were assessed by the responses to the questionnaire. A total of 6391 respondents met the eligibility requirements [22]. The study data is a publically avaliable open data set, and all methods were carried out in accordance with the local university’s guidelines and regulations for use of Human data.

### Outcome measures

To assess the subjective stress of COVID-19, the items of the Impact of Event Scale-6 (IES-6) were adapted (Supplementary Table 1, Additional file 1). The test items consisted of a 4-point Likert scale ranging from 0 to 3 (0 = not at all; 1 = several days; 2 = more than half the days; 3 = nearly every day). The summed score ranged from 0 to 18, with higher scores indicating greater PTSD symptoms.

The IES-6 [23] is a 6-item short version of the Impact of Event Scale-Revised (IES-R) [24] that measures the principal components of PTSD [18]. The IES-6 demonstrated good sensitivity (*r* = .88) and specificity (*r* = .85) with a standard PTSD semi-structured interview conducted by physicians [20, 25].

### Data analysis

Of the total subjects (*n* = 6391), 600 random samples (approximately 10%) were analyzed. Descriptive statistics were used to examine participants’ demographic characteristics. Unidimensionality was examined using principal component analysis (PCA) of Rasch residuals. In addition, once the instrument revealed a single dominant measurement structure, we conducted item-level analysis using the Rasch model, including rating scale analysis, item fit statistics, precision, differential item functioning (DIF), and construct validity. Statistical analyses were performed using SAS v. 9.4 and Rasch analysis was performed using Winsteps v. 4.7.1.

### Unidimensionality

#### Principle component analysis (PCA) of Rasch residuals

Principal component analysis (PCA) of the residuals was used to examine the unidimensionality assumption in the test items [26]. Unlike conventional factor analysis, PCA of Rasch residuals is performed after excluding the target configuration, and secondary dimensions are detected. Unidimensionality assumes that the eigenvalue for the first contrast is less than 2.0 or that the variance ratio explained by the measurement is greater than 20% [27].

#### Local independence

Local independence means that when the structure level is controlled, the response to the item is not related to another item. To identify local independence, the residual correlation matrix was examined [28]. An average item residual correlation exceeding .20 was interpreted as indicating dependency [29].

### Rasch analysis

#### Rating scale analysis and item fit statistics

The rating scale model was applied to the six items of the IES-6. To examine the fit of the data to the Rasch model, a rating scale analysis was used. We determined the extent to which the empirically obtained data matched the predictions of the model.

##### Rating scale analysis criteria

1) at least ten observations in each rating scale, 2) monotonically advanced average measure in rating scale categories, and 3) outfit mean squares (MnSq) less than 2.0 for rating scale categories [30].

##### Item fit

We used the mean square residual (MnSq) and standardized mean square residual (Zstd) to examine item fit. MnSq values between .6 and 1.4 and Zstd values between − 2.0 and 2.0 indicate acceptable fit [31]. It is agreed that up to 5% of the sample could demonstrate misfit without being a serious threat to validity [32]. The values provided by this model are expressed in the logit scale, which is a logistic transformation of the observed scores with a mean of 0 and a standard deviation of 1. According to construct theory, suitable items can measure intended unidimensionality and Rasch analysis is a powerful tool for evaluating construct validity [33]. The conditional maximal likelihood estimator (CMLE) was used for the parameter consistency [34].

#### Validity

##### Construct validity

The sequence of reasonable or conceptual item difficulties for the assessment item is interpreted as construct validity [33]. In the Rasch model, the difficulty of the evaluation items and the IES-6 score are located in the same linear continuum (logit), and the matching between the evaluation items and the human measurement is presented by the Wright map. We analyzed whether the sequence of difficulty layers of the estimated evaluation items in the Rasch model matched the logical progression from the easiest to the most challenging. Furthermore, we examined whether ceiling and floor effects were at least 5% of the samples in measurements with the maximum and minimum criteria [35].

##### Convergent validity

To secure convergent validity, the person measure was correlated with the PHQ-4, which assesses anxiety and depression symptoms. Spearman correlation analysis was used to examine the correlation between PHQ-4 and the IES-6 scores.

#### Differential item functioning (DIF)

In Rasch and item response theory models, the probability of item responses should be a function of the basic characteristic level of people [36]. We conducted a DIF to examine the linear invariant estimation of item difficulty parameters based on the Rasch model [37]. If different group members have the same characteristic level but different response probabilities, the entry represents differential item functioning (DIF). We used the Rasch-Welch t test to compute the size of the DIF [38]. The following are the effect criteria for DIF: (a) a moderate to large DIF (greater than .64 logits in the DIF contrast, thus indicating the difference in item difficulties between the two comparison groups) and (b) a slight to moderate DIF (greater than .43 in the DIF contrast). The significance of DIF contrast was determined at an alpha value of .05 with a two-sided Rasch-Welch *t-*test [38].

#### Precision

Personal reliability reflects the degree of impact of measurement scores on measurement errors [39]. In Rasch analysis, every participant is given a Rasch score with an individual person reliability. Personal reliability used the sum score of IES-6 for group comparisons: a score of .7 or higher was considered suitable and .9 or higher was suitable for comparing individual reliability [40]. A person separation index of 2.00 indicates acceptable levels of separation, where a value of 3.00 represents a good separation level. We calculated MacDonald’s omega to examine the reliability of the instrument [41].

## Results

### Demographic characteristics

The demographic characteristics of participants are displayed in Table 1. The sample comprised 600 participants from the COVID-19 online survey. Females accounted for 59% of the participants (*n* = 354). The highest percentage of respondents were in their 50s, followed by those in their 60s, 40s, and 30s. A majority 62.7% (*n* = 376) were employed. A total of 29.7% (*n* = 178) of participants had children under the age of 18 and 70.3% (*n* = 422) had no children. There were no significant differences between the total and sample groups.

**Table 1:** Demographic characteristics of participants

Variable	Sample, *n* (%)
Sex	
Female	354 (59.0)
Male	244 (40.7)
Other/No disclosure	2 (.3)
Age group (years)	
18–29	59 (9.8)
30–39	86 (14.3)
40–49	108 (18.0)
50–59	187 (31.2)
60–69	121 (20.2)
≥ 70–79	39 (6.5)
Residence type	
Rural	184 (30.7)
Suburban	313 (52.2)
Urban	103 (17.2)
Employment status	
Working	376 (62.7)
Unemployed	80 (13.3)
Student/Retired/Unpaid worker (e.g., homemaker, eldercare, childcare)	144 (24.0)
Work in healthcare/clinical setting	
Yes	100 (16.7)
No	500 (83.3)
Has children under 18	
Yes	178 (29.7)
No	422 (70.3)
Educational attainment	
High school or below	94 (15.7)
Some college	205 (34.2)
Bachelor’s degree	175 (29.2)
Masters/professional degree or above	126 (21.0)

### Rasch analysis

#### Unidimentionality

The Rasch model explained 54.1% of the raw variance in PCA of Rasch residuals. The eigenvalues of the first, second and third contrast were 1.43 (11.0%), 1.32 (10.1%) and 1.09 (8.4%), respectively. The average of residual correlation was .19 (< .20).

#### Rating scale analysis

The IES-6 for COVID-19 utilizes a 4-point Likert scale, and the results of checking the suitability of the categories are listed in Supplementary Table 2, Additional file 1. According to the rating scale analysis, the four response categories were all selected more than ten times, had a good fit (outfit MnSq ranges .82–1.08), and average measures and Andrich thresholds (step calibration) were increased sequentially.

#### Fit statistic

The infit and outfit of the Rasch model were checked to determine the goodness of fit of the items of the IES-6 for COVID-19. Most of the items were found to be suitable, but in item 5 (“I tried not to think about the coronavirus”; MnSq = 1.63, Zstd = 9.05), MnSq was found to be greater than 1.4 and Zstd was greater than 2.0, which made it a misfitting item. However, the contents of item 5, indicating avoidance of stress-causing factors, have not been deleted as they are the main clinical aspects of PTSD [42]. Table 2 presents the item-fit statistics.

**Table 2:** Item fit, Item difficulty hierarchy results

Item number	Measure (logits)	Model	Infit		Outfit	
		SE	MnSq	Zstd	MnSq	Zstd
1	−.53	.06	.76	−4.43^b^	.74	−4.25^b^
2	−.83	.06	1.12	2.00	1.20	2.66^b^
3	−.31	.06	.79	−3.93^b^	.80	−3.33^b^
4	.86	.06	.93	−1.11	.80	−2.90^b^
5	.20	.06	1.63^a^	9.05^b^	1.70^a^	9.18^b^
6	.60	.06	.94	−1.00	.88	−1.88

*Note*. *MnSq* mean square, *Zstd* standardized mean square

^a^MnSq value less than .6 or greater than 1.4, ^b^Zstd value less than − 2.0 or greater than 2.0

The percentage of misfitting persons was 7.33% (*n* = 44), but the item parameter was invariant even if the misfit individuals were removed.

#### Validity

##### Construct validity

**Fig. 1:**
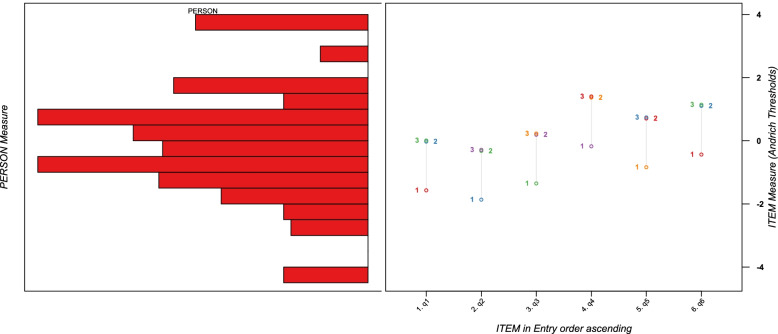
Wright map

The item difficulty hierarchy of the assessment items is presented in Table 2. Item 4 was the most difficult item, and Item 2 was the easiest. In addition, the difference between the item average and the person average was less than .5 logits. Left of Fig. 1 indicated distribution of person ability, and right showed item difficulty. Person ability scores were distributed between − 4 and 4, and items were placed within this range. The number of people who received the maximum score was 47 (7.8%) and the minimum score was 23 (3.8%). The maximum was slightly greater than 5%, but it can be considered to have no ceiling or floor effect (Fig. 1).

##### Convergent validity

To assess convergent validity, we calculated the correlation with the PHQ-4 total score, the tool included in the same database. The PHQ-4 was used to evaluate anxiety and depression symptoms among people who experienced the COVID-19 pandemic. Spearman’s correlation revealed a significant correlation between the two assessment tools (*r* = .109, *p* = .007).

#### DIF

Following prior studies, the DIF contrast of age, sex, residence type, working status, having children under the age of 18, and healthcare or clinic of profession were identified as indicative of PTSD involvement [43, 44]. The age group was divided into two groups based on the age of 50 years, based on a previous study by Karatzias et al. [43], which suggested that there was a significant difference in the number of people diagnosed with PTSD related to COVID-19. Item 5 showed significant DIF with or without children under 18 (DIF contrast = 0.47, *p* = .0003; Table 3). There were no significant differences in the DIF among the other variables.

**Table 3:** Differential item functioning across having children under the age of 18

Item number	Having children under 18 years old (No vs. Yes)				
	DIF	Joint	Rasch-Welch		
	Contrast	SE	*t*	*df*	Prob.
1	−.26	.13	−2.04	270	.04
2	−.17	.13	−1.33	267	.19
3	.21	.12	1.73	275	.08
4	−.07	.13	−.56	289	.58
5	.47^a^	.13	3.71	272	.00^*^
6	−.21	.13	−1.62	289	.11

*Note*. Reference group = Having children under 18 years old, DIF = differential item functioning

^a^Absolute value of DIF contrast over .43

^*^p < .001

#### Precision

The IES-6 for COVID-19 had an acceptable person reliability of .76. In addition, the person separation index was 1.60, 2.47 person strata, which is an acceptable level. The MacDonald’s omega coefficient was .91.

## Discussion

The IES-6 has been widely used as a tool for measurement of subjective stress [23]. However, it was necessary to ensure that this tool was also suitable for the measurement of post-traumatic stress induced in the special situation of the COVID-19 pandemic. This study aimed to verify the validity of the items and rating scale categories of the IES-6 adjusted for COVID-19. The IES-6 showed validity in all items except item 5, and the response category was also appropriate.

Rasch analysis was used to validate the results. The eigenvalue, derived from the PCA of Rasch residuals, indicated that this tool satisfies the unidimensional assumption of Rasch analysis. In addition, local independence between items was identified by the average of the residual correlation. It means that the six items of the IES-6 explain one factor, which we refer to as “subjective stress of coronavirus.” When these assumptions are satisfied, it can be seen that the Rasch model theory can be applied [26]. This process is different from confirming the validity using the 3-factor model, as seen in another study [19]. The difference in traumatic experience, i.e., the bank robbery, seems to have had an effect.

The IES-6 had a logical hierarchy of each item’s disparity; item 4 was the most difficult and item 2 was the easiest. Owing to the strong transmission power of the coronavirus, it is possible that most of the participants chose a low score on item 2 (“I felt watchful or on-guard”). In this context, the respondents may have given high scores on item 4 (“I was aware that I still had a lot of feelings about the coronavirus, but I didn’t deal with them.”) because it is difficult to deal with the idea of the virus in a situation where personal and social COVID-19 prevention continues [4]. Fig. 1 is a Wright map that visualizes the hierarchy of these items. In the map, item difficulty and person ability are similarly distributed. Through the distribution of person ability and the item difficulty, it was found that IES-6 could measure the overall level. Matching between the two would reduce the likelihood of errors that could occur in personal measurement estimation [45].

Both ceiling and floor effects were acceptable and did not threaten the validity of the evaluation tool. In addition, the five items had goodness-of-fit and there was no DIF. However, item 5 (“I tried not to think about the coronavirus”) was found to be a misfit item through fit statistics, and the estimated average was higher than those without children. These values were consistent with those of other studies, which show that people pay more attention to the virus when they have children [43]. This item is important because it represents an important clinical characteristic indicating the avoidance of the cause of stress, one of the major symptoms of PTSD screening [42]. In addition, if the item was deleted, person reliability was degraded and could not be removed.

When item 5 was not excluded, person reliability and person separation indexes indicated high precision in measuring coronavirus-related stress. MacDonald’s omega coefficient was also acceptable. The scores of the PHQ-4 and IES-6 included in the same questionnaire were statistically significant, and consistent with those of previous studies, which indicate that people had the higher stress level showed the higher depressive and anxiety level [20]. This correlation shows the convergent value of IES-6.

### Limitations

The number of items on the IES-6 was only six, and this number of items may seem too few to meet the diagnostic criteria for PTSD. Since the nature of secondary data analysis, we were not able to add or modify the items used for collection of the data. In addition, the IES-6 has widely used to screen of PTSD symptoms. So, despite of the small number of the test items, it can be used as a screen tool for assessing subjective stress. Second, the participants in this study consisted only of adults residing in U.S. who could access Facebook. These restrictions prevent adolescents and individuals from other cultures that would allow us to generalize whether the IES-6 is appropriate. In addition, it was not possible to measure the mental health of individuals with severe disabilities, who could not use Facebook. The COVID-19 pandemic is known to have had a greater impact on them and has a longer-term impact on their mental health [46]. Therefore, it is necessary to collect and analyze the results through a wider survey in the future. Third, due to the nature of the online survey, there is a possibility that answers will not always be accurate, so the ratio of misfit persons was slightly higher. However, it can be considered that misfit persons did not affect this result because there was no difference even when they were deleted. Finally, it is important to pay attention to the interpretation of the results because item 5 had a misfit but could not be deleted. After that, it is necessary to supplement and improve the questions so that PTSD can be selected more accurately.

## Conclusion

Despite the misfitting of one item, the results of this study indicate that the IES-6 is acceptable for measurement of stress in American adults, who Have experienced the COVID-19 pandemic. The IES-6 for COVID-19 showed acceptable psychometric properties in unidimensionality, rating scale, fit statistics, DIF, and precision. COVID-19-related stress is a problem that affects many aspects of an individual’s daily life. Therefore, rapid screening and appropriate approaches are needed, and it is suggested that the use of the IES-6 can be useful in this process.

## Supplementary Information

**Additional file 1: Table S1.** Items of the IES-6 for COVID-19. **Table S2.** Rating scale analysis of the IES-6 for COVID-19.

## Data Availability

The dataset supporting the conclusions of this article is available in the [Inter-university Consortium for Political and Social Research (ICPSR)] repository, [unique persistent identifier and hyperlink to dataset in https://www.openicpsr.org/openicpsr/project/120308/version/V1/view].
