# Supplementary material for: Impact of Event Scale-6 (IES-6) for U.S. adults who experienced the COVID-19 pandemic
Source: BMC Psychiatry. 2022 Jul 22;22:490. doi: 10.1186/s12888-022-04136-2 (PMC9305040; doi:10.1186/s12888-022-04136-2)
Supplement: Supplementary file 1 — Additional file 1: Table S1. Items of the IES-6 for COVID-19. Table S2. Rating scale analysis of the IES-6 for COVID-19. [file 12888_2022_4136_MOESM1_ESM.docx]

**Additional file 1**

**Supplementary Table 1**

*Items of the IES-6 for COVID-19*

| Number | Descriptions |
| --- | --- |
| 1 | I thought about the coronavirus when I didn’t mean to. |
| 2 | I felt watchful or on-guard. |
| 3 | Other things kept making me think about the coronavirus. |
| 4 | I was aware that I still had a lot of feelings about the coronavirus, but I didn’t deal with them. |
| 5 | I tried not to think about the coronavirus. |
| 6 | I had trouble concentrating. |

**Supplementary Table 2**

*Rating scale analysis of the IES-6 for COVID-19*

| Score | Count | % | Infit  (MnSq) | Outfit  (MnSq) | Andrich threshold | Average measure |
| --- | --- | --- | --- | --- | --- | --- |
| 0 | 1,028 | 29 | 1.10 | 1.14 |  | (-2.29) |
| 1 | 974 | 27 | .86 | .82 | -1.04 | -.53 |
| 2 | 562 | 16 | .83 | .82 | .50 | .67 |
| 3 | 1,036 | 29 | 1.14 | 1.18 | .54 | (2.06) |

Note. MnSq = mean square
